# An Intermittent Live Cell Imaging Screen for siRNA Enhancers and Suppressors of a Kinesin-5 Inhibitor

**DOI:** 10.1371/journal.pone.0007339

**Published:** 2009-10-05

**Authors:** Melody Tsui, Tiao Xie, James D. Orth, Anne E. Carpenter, Stewart Rudnicki, Suejong Kim, Caroline E. Shamu, Timothy J. Mitchison

**Affiliations:** 1 Department of Systems Biology, Harvard Medical School, Boston, Massachusetts, United States of America; 2 ICCB-Longwood Screening Facility, Harvard Medical School, Boston, Massachusetts, United States of America; 3 Broad Institute of Harvard and MIT, Cambridge, Massachusetts, United States of America; University of Edinburgh, United Kingdom

## Abstract

Kinesin-5 (also known as Eg5, KSP and Kif11) is required for assembly of a bipolar mitotic spindle. Small molecule inhibitors of Kinesin-5, developed as potential anti-cancer drugs, arrest cell in mitosis and promote apoptosis of cancer cells. We performed a genome-wide siRNA screen for enhancers and suppressors of a Kinesin-5 inhibitor in human cells to elucidate cellular responses, and thus identify factors that might predict drug sensitivity in cancers. Because the drug's actions play out over several days, we developed an intermittent imaging screen. Live HeLa cells expressing GFP-tagged histone H2B were imaged at 0, 24 and 48 hours after drug addition, and images were analyzed using open-source software that incorporates machine learning. This screen effectively identified siRNAs that caused increased mitotic arrest at low drug concentrations (enhancers), and vice versa (suppressors), and we report siRNAs that caused both effects. We then classified the effect of siRNAs for 15 genes where 3 or 4 out of 4 siRNA oligos tested were suppressors as assessed by time lapse imaging, and by testing for suppression of mitotic arrest in taxol and nocodazole. This identified 4 phenotypic classes of drug suppressors, which included known and novel genes. Our methodology should be applicable to other screens, and the suppressor and enhancer genes we identified may open new lines of research into mitosis and checkpoint biology.

## Introduction

Kinesin-5 (also known as Kif-11, Eg5 and KSP), is a plus-end-directed, tetrameric motor protein required for establishing spindle bipolarity during mitosis [1]–[4]. The first small molecule Kinesin-5 inhibitor (K5I) was identified in a cell-based screen for mitotic arrest[5]. Potent and specific K5Is were then developed in the hope of anti-cancer drugs that were as effective as Vinca alkaloids and taxanes, but lacked their neurotoxicity [6]. Cancer cells treated with K5Is arrest in mitosis with a monopolar spindle, and subsequently undergo cell death by the intrinsic apoptosis pathway [7]. Although all cancer cell lines tested arrest in mitosis when treated with K5Is, the fraction of cells that undergo apoptosis varies greatly for unexplained reasons [8]–[10]. In clinical trials, as hoped, K5Is do not cause neurotoxicity, but they do cause severe bone marrow toxicity, and it is not yet clear which patients, if any, will benefit from treatment [6]. To facilitate success of these drugs, it will be necessary to discover effective combination therapies, and/or identify particular cancer genotypes that respond well. This will require deeper understanding of cell responses. To this end, we sought to identify genes for which partial or full loss of function makes cells either more resistant (suppressors) or more sensitive (enhancers) to drug treatment.

RNA interference (RNAi) technology provides an efficient strategy to systematically test the role of individual genes in the response of live cells or model organisms to drug treatments [11]–[13]. However, most RNAi screens in human or *Drosophila* cells have used assays where cells are fixed or lysed at a certain time point to obtain a readout, which limits the amount of data that can be obtained. RNAi screens with live cell imaging readouts have been reported [14], but these require complex equipment and analysis software. Here, we report a simple intermittent live cell imaging method for scoring cell cycle and cell death phenotypes in living cells, and its use to find suppressors and enhancers of a Kinesin-5 inhibitor. We used this method to screen a library of siRNAs targeting the full human genome, and further characterized the strongest suppressors using time-lapse imaging. We found several expected genes, and others that may reveal new cellular systems involved in how the mitotic spindle responds to drug perturbation.

## Methods

### Cell culture

HeLa H2B-GFP cells [15] were grown at 37°C under 5% CO_2_ in Dulbecco's Modified Eagle Medium supplemented with 10% fetal calf serum and 1% penicillin streptomycin (Gibco). The doubling time of this HeLa H2B-GFP cell line is approximately 18 hours. Cells were grown to 80–90% confluency in 75 cm^2^ flasks and passaged every two days. Cells were frozen down in multiple aliquots at passage 3 to 7, and stored in liquid nitrogen until use. Only cells with passage numbers less than 15 were used for screening. For siRNA transfection, 2500 cells/well were plated in 384 well plates (Corning) using a Matrix WellMate. Under these conditions the cells reach 60–70% confluency after 24 hours.

### Human Genome siRNA Libraries

Two Dharmacon siRNA SMARTpool libraries were used for primary screening. Both were arrayed such that each library well contained one pool of four siRNA duplexes directed against one gene. A smaller library of 509 SMARTpools that covered most of the kinases in the human genome (Dharmacon siARRAY siRNA Kinases Library Thermo Fisher Scientific, Lafayette, CO) was provided as a generous gift by Pfizer Inc. (Groton CT) and was originally obtained from Dharmacon in the late fall of 2004. This was used mainly to optimize procedures, though we did recover some kinases as enhancer hits. A full human genome library of 21,121 siRNA SMARTpools, (Dharmacon siARRAY siRNA Library, Human Genome, G-005000-05, Thermo Fisher Scientific, Lafayette, CO) was obtained by the Harvard Medical School ICCB-Longwood Screening Facility in 2006. This was used for the full genome screen.

All library stocks were made up in 1x Dharmacon Buffer (20 mM KCl, 6 mM HEPES-pH 7.5, 0.2 mM MgCl2), transferred to twin.tec 384-well PCR plates (Eppendorf), sealed with PlateLoc Thermal Seals (Velocity-11), and stored at −20C. Concentrated Master Stocks of the siRNA libraries are stored at 10 uM or 20 uM. Aliquots from the Master Stocks were diluted in 1x Dharmacon Buffer to make 1 uM Screening Stocks of the libraries, which were also stored in twin.tec 384-well plates at −20C.

**Figure 1:**
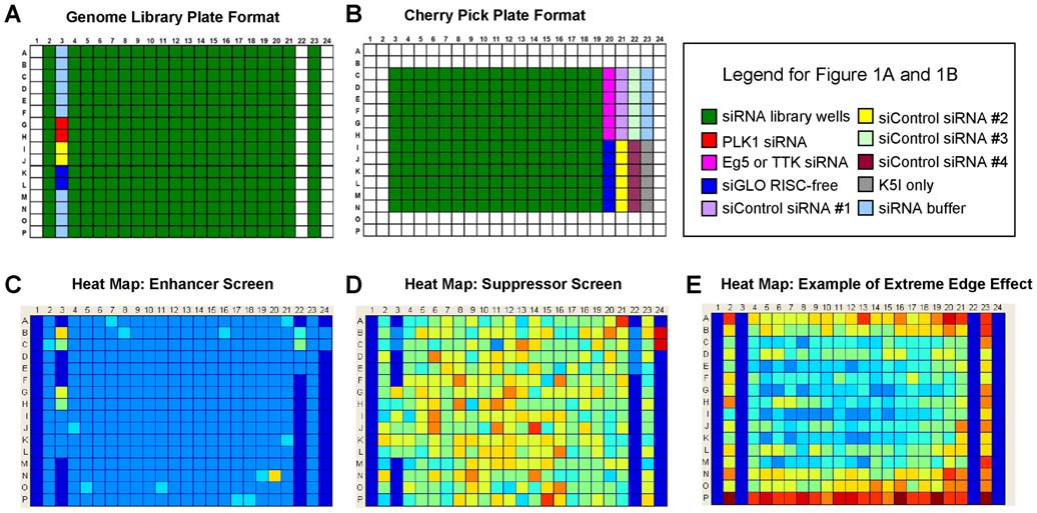
siRNA library and cherry pick source plate layouts. (A) siRNA library pools were plated in twin.tec 384 well plates with controls in column 3 (see key). (B) A typical cherry pick source plate for the KI5 enhancer and suppressor screens. Individual duplexes were plated randomly (one duplex per well) in twin.tec 384 well plates so that duplexes targeting the same gene were unlikely to lie adjacent to each other. The edge wells (two outside rows and two outside columns) were left empty in each cherry pick source plate. Control siRNA duplexes were plated in columns 20–22 (see key). (C) Heat map of enhancer screen plate after 48 hours using ratios of monopolar spindles to interphase cells; wells with a higher ratio are displayed as warmer colors (yellow), allowing visual identification of potential hits (D) Heat map of suppressor screen plate using ratios of monopolar spindles to total object count; the cooler color (blue) indicates a positive well. (E) Heat map showing an extreme example of edge effects in a sample plate from the enhancer screen.

Control siRNAs were plated in duplicate in each library plate. Polo-like kinase 1 (PLK1, Dharmacon M-003290-01) was used as a positive control for mitotic arrest and apoptosis. Negative controls included siCONTROL #2(Dharmacon D-001206-13-05) non-targeting siRNA and siGLO RISC-Free siRNA (Dharmacon D-001220-01-05) to control for non-sequence-specific effects. A diagram of the plate layout for a typical ICCB-Longwood siRNA library stock plate is shown in Figure 1A.

Re-testing of primary screening positives was carried out using the four individual siRNA duplexes that comprise each siRNA SMARTpool. For initial confirmation, these duplexes were obtained from the ICCB-Longwood human genome siRNA duplex library (Dharmacon Human Genome siRNA Library, Thermo Fisher Scientific, Lafayette, CO, obtained in 2006), which comprises the approximately 84,000 individual siRNA duplexes corresponding to the 21,121 human genome SMARTpools. Individual duplexes are stored in Master Stocks at 10 uM or 20 uM, and in Cherry Pick Stocks at 1 uM, in twin.tec 384-well plates. Duplexes were picked from Cherry Pick Stock plates using an EVO75 (Tecan) liquid handler served by a BenchCel plate stacker (Velocity11) and arrayed in cherry pick source plates for screening. A diagram of the cherry pick plate layout used in both the enhancer and suppressor screens is shown in Figure 1B.

### Screening Protocol

**Figure 2:**
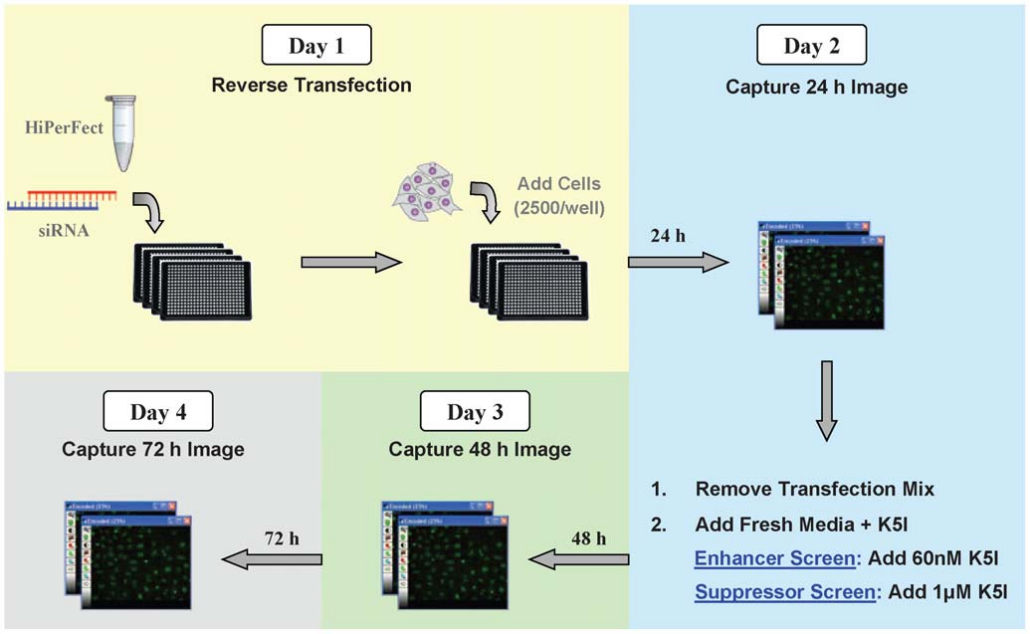
Schematic of primary screen assay protocol. Day 1: HeLa H2B GFP cells are transfected with siRNAs. Day 2: 24 hours after transfection, cells are imaged using a screening microscope. Transfection mix is removed, and media containing 60 nM Kinesin 5 inhibitor (K5I; Enhancer screen) or 1 uM K5I (Suppressor screen) is added. Days 3 and 4: 48 and 72 hours after transfection, cells are imaged to monitor phenotypic changes.

Figure 2 shows a flowchart of the screening protocol. Screens of SMARTpool libraries for the primary screens and of selected individual duplexes for secondary screens were carried out following essentially the same protocol, except for the transfection step and the statistical analysis of each plate, as noted below.

### siRNA Transfection—Primary screen

Cells were reverse transfected with each siRNA SMARTpool at a final concentration of 24 nM using HiPerFect transfection reagent (Qiagen). Automated transfections were carried out in a Bioprotect II biosafety cabinet (Baker) using a Velocity11 Bravo liquid handler for pipeting. A Wellmate (Matrix) was used for plate filling. Each siRNA pool was diluted to 429 nM by mixing 3 ul of 1 uM siRNA Screening Stock with 4 ul of OptiMEM I Reduced Serum Medium 1x (Invitrogen) in an intermediate 384-well twin.tec plate. Next, 3 ul of each diluted siRNA pool was added to 9.6 ul of OptiMEM and 0.4 ul of HiPerFect transfection reagent previously plated in each well of in a black, clear bottom-384 well assay plate (Corning). Transfection reagent and siRNAs were allowed to complex at room temperature for 10 minutes. HeLa H2B GFP cells (2500 cells per well in 40 uL of DMEM) were then added to the HiPerFect + siRNA complexes and incubated at 37°C and 5% CO_2_ for 24 hours.

siRNAs targeting the genes KIF11 and TTK were used as positive controls in the enhancer and suppressor screens, respectively. Negative controls included Dharmacon si*CONTROL* Non-targeting siRNA #1, #2, #3, and #4 (Thermo Fisher Scientific catalogue numbers D-001210-01-05, D-001210-02-05, D-001210-03-05, D-001210-04-05) to control for non-sequence specific events and Dharmacon siGLO RISC-free siRNA (Thermo Fisher Scientific) as a transfection control.

### siRNA transfection—Individual duplexes for secondary screening

Cells were transfected with individual siRNA duplexes at a final concentration of 18 nM using HiPerFect transfection reagent, following a reverse-transfection protocol. Control siRNAs were plated in sextuplicate in each assay plate in Columns 20–23 as shown in Figure 1B. With the exception of these changes in siRNA concentration and plate layout, transfection of individual duplexes was carried out with the same protocol as the primary screen.

### Kinesin-5 Inhibitor Treatment

EMD534085, a potent and specific small molecule Kinesin-5 inhibitor (K5I) that targets the mitotic kinesin Eg5/KIF11 was used in this study[8], [9]. The biological effects of this K5I are similar to that of previously described potent and specific Kinesin-5 inhibitors[3], [7], [9]. Cells were imaged 24 hours after transfection to provide baseline data on cell density. After imaging, the transfection mix/media was removed by aspiration with a 24 channel straight manifold (Drummond Scientific) and replaced with 50 uL of fresh media (Dulbecco's Modified Eagle Medium with 10% fetal calf serum and 1% penicillin streptomycin) containing 60 nM K5I for the enhancer screen or 1 uM K5I for the suppressor screen. After addition of K5I, cells were incubated for an additional 48 hours and imaged for phenotypic changes at 24 hour intervals.

### Microscopy

Images were acquired on an ImageXpress Micro high content screening microscope (MDS Analytical Technologies, formerly Molecular Devices) with a Photometrics CoolSNAP ES digital CCD camera and a 20x Plan Fluor ELWD objective. Four single wavelength GFP images (488 nm excitation, FITC filter cube, Semrock) were taken in each well to capture 200–600 cells per well. HeLa H2B-GFP cells were imaged without fixation. No environmental chamber was used with the ImageXpress Micro. Assay plates were kept in the incubator until imaging time and transported to the microscope in an insulated bag. On average, each assay plate was imaged at room temperature for 20 minutes every 24 hours. Samples were illuminated for 100 ms per image to obtain the optimal signal to noise ratio for automated phenotyping. The 100 ms imaging time at 488 nm is not long enough to significantly damage cells, as cell damage typically occurs at high intensities of Ultraviolet radiation (350 nm) [2], [16]. Each well was imaged at 3 time points: the 24H Image was taken 24 hours after transfection and before K5I addition in order to obtain a baseline cell count for each siRNA condition. The 48H and 72H Images were taken 48 hours and 72 hours after transfection.

### Image Analysis

We analyzed acquired images with CellProfiler, an open-source image-analysis package [17]. For image segmentation, we applied a modified version of the Ridler-Calvard method [18] (referenced as the Adaptive Ridler-Calvard in CellProfiler) to determine the threshold. This method takes into account the threshold variation across the image. Although the nuclei are evenly dispersed in most images, clustered nuclei occasionally appear. Therefore a second step was taken to divide the clustered objects using a watershed segmentation[19], and all cells bordering the edge of images were discarded. Over 80 different shape, intensity and texture features were measured for each cell.

Each nucleus was classified as interphase, monopolar, or “other” (neither interphase nor monopolar). For phenotypic classification, we took a machine learning approach using CellProfiler Analyst, an extension of CellProfiler [20], [21]. For each phenotype, an initial training set was manually built. About 1000 cells were randomly retrieved from the entire dataset in batches and manually sorted into positive and negative training sets until training process reached convergence.

Heat maps were used to visualize the data for quality control purposes. For the enhancer screen, heat maps were plotted for the ratio of cells in monopolar arrest to cells in interphase. Wells with a higher ratio are displayed as warmer colors (yellow, in Figure 1C), allowing us to identify potential hits, and also to assess potential plating artifacts. Heat maps for the suppressor screen were plotted using the ratio of monopoles to total objects, since these data included more cells with abnormal nuclei that were not clearly scored as interphase. A cooler color (blue, Figure 1D) indicates a positive well. Plotting heat maps also allowed us to see artifacts resulting from edge effects or uneven plating (e.g. see Figure 1E for an extreme example) that could be corrected in analysis.

### Statistical Analysis of Imaging Data from Primary Screen

Both interphase and monopolar cells were classified and counted at 48 hours and 72 hours after transfection. The phenotypic counts were used to calculate enhancer or suppressor scores for each well, which were then ranked to identify the siRNA pools that were positive in each assay. An evaluation of the total cell count in each well at 24 hours was also included in the score formula to eliminate wells with extremely low cell density.

#### Enhancer Screen

**Figure 3:**
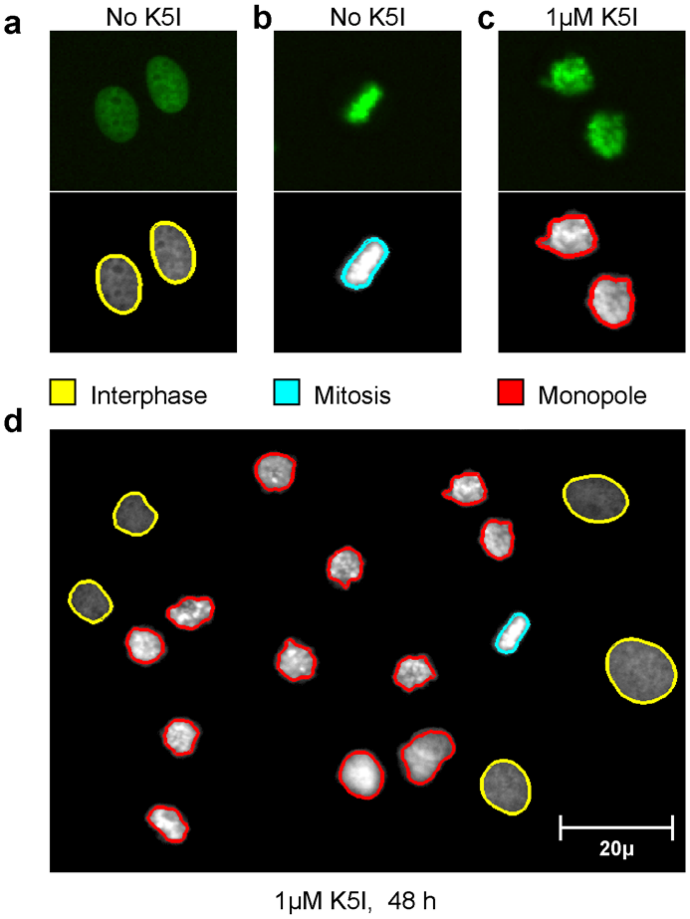
Examples of chromosomal phenotypes in the screening assay. All cells were grown in 384-well plates and treated according to the primary screen assay protocol outlined in Figure 2 and described in Materials and Methods. These samples were all transfected with a non-targeting siRNA duplexes. (A) Interphase cells and (B) Mitotic cells selected from control wells undergoing normal cell cycle progression; no Kinesin 5 inhibitor (K5I) added. (C) Monopolar spindles formed in the presence of 1 uM K5I. (D) Analysis of cells treated with 1 uM K5I after 48 hours.

**Figure 4:**
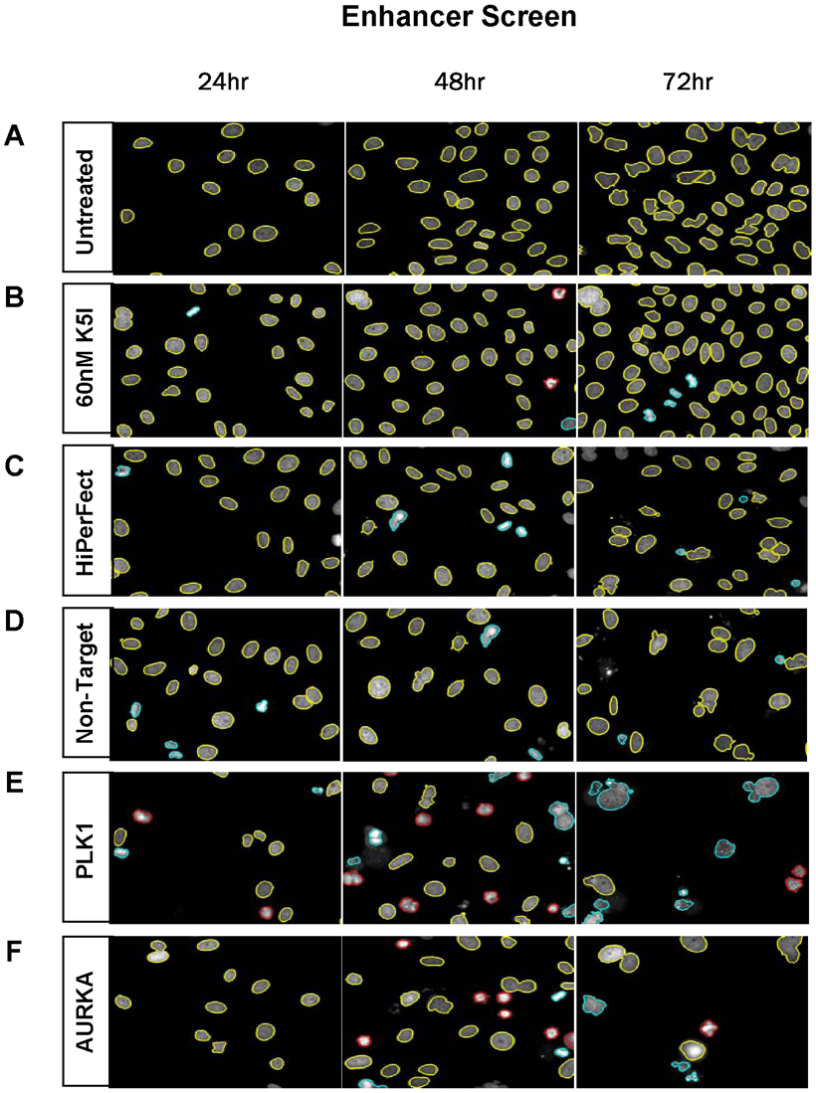
Sample strong hit phenotypes from the K5I Enhancer screen. The nuclei from each time point are color-coded to illustrate their CellProfiler classifications: yellow = interphase, red = monopolar, blue = unclassified objects. As illustrated in Fig. 2, transfection occurred at T = 0. (A) untreated HeLa H2B-GFP cells, (B) untransfected cells treated with 60 nM K5I, (C) mock transfected cells treated with HiPerFect transfection reagent and no K5I. In panels D–F, 60 nM K5I was added to cells 24 hours after transfection with (D) non-targeting siRNA, or siRNAs directed against (E) PLK1 or (F) AURKA.

For an siRNA to score as positive in the enhancer screen, it must cause a significant increase in the ratio of monopolar to interphase nuclei (color-coded as red and yellow objects in Figures 3 and 4) at 48 and 72 hour time points relative to the population average in a single experiment. A single experiment consists of all plates transfected on the same day. The large experiment-to-experiment variation makes the comparison across the whole genome dataset biased towards the experiments with higher transfection efficiency and lower toxicity.

The enhancer score is defined as
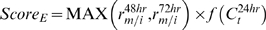


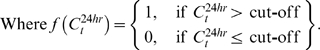


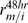
 and 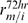 are the monopolar to interphase nuclei ratios at the 48 and 72 hour time points. The value 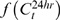 is added to discard wells with significantly low starting cell densities, characterized by the total cell count at the 24 hour time point, 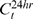 (24H Image). The cut-off for this step function was arbitrarily chosen to be two standard deviations below the mean of the population. The resulting enhancer score is directly proportional to siRNA effectiveness in enhancing cell response to K5I.

#### Suppressor Screen

For an siRNA to score as positive in the suppressor screen, there must be a significantly lower percentage of monopolar arrested nuclei at 48 and 72 hours after transfection. Due to the high ratio of drug-induced apoptosis in the suppressor screen, fragments of DNA from dead cells often float on top of healthy interphase nuclei. Hence, we used a monopole to total nuclei count ratio to calculate the suppressor score. All statistics were calculated relative to population average of cells imaged at the same time points from the same experiment.

The suppressor score is defined as
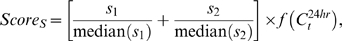


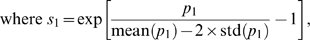


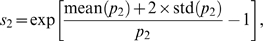


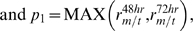


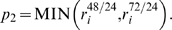



The 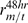 and 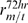 terms are the monopolar to total nuclei count ratios at the 48 and 72 hour time points. The values 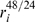 and 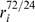 characterize the cell survival rate and represent the ratio of interphase nuclei at the 48 and 72 hours in relation to the 24 hour time point. Both *p*_1_ and *p*_2_ are exponentially rescaled and normalized before addition to give the suppressor score. The 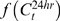 step function was also introduced in the suppressor screen, discarding wells with low cell density. The suppressor score is inversely proportional to siRNA effectiveness in suppressing cell response to K5I.

### Statistical Analysis of Imaging Data from Secondary Screens—Individual duplexes

For re-testing potential hits in secondary screens, a new baseline was established to account for enrichment of positively scoring wells. Multiple non-targeting siRNA oligos were placed as negative controls on each cherrypick source plate (Figure 1B). The resulting data was used to calculate the mean and standard deviation for negative controls. We observed a large variation within the negative controls in the enhancer screen for unknown reasons, presumably related to the threshold drug-concentration. As a cut-off, we scored as positive any duplex with a monopole to interphase ratio of one standard deviation or more above the mean. Re-testing data for the suppressor screen, where the drug was used at a saturating concentration, was less noisy. Here, any duplex with a monopole to total nuclei ratio at least two standard deviations below the mean scored positive.

### Long-term Timelapse Microscopy of Suppressor Hits

HeLa H2B-GFP cells were reverse transfected with individual siRNA duplexes or pools (both at 15 nM final concentration) in 96 well glass bottom plates (MatTek P96G-0-5-F). 24 hours after transfection, K5I was added to the cells, so that the final inhibitor concentration was 1 uM. To prevent evaporation during imaging, 50 uL of mineral oil was added to each well. Starting immediately after drug and mineral oil addition, the cells were imaged at 10 minute intervals for 48 hours on an automated inverted fluorescence microscope (Nikon TE2000E) fitted with an incubation chamber and automated focus. Both GFP and phase contrast images were collected. The chamber was kept at 37°C with 5% CO2 throughout imaging. For each siRNA pool and duplex reagent tested, 50–100 cells were scored visually for phenotypic changes during imaging.

### Taxol and Nocodazole Assays

HeLa H2B-GFP cells were reverse transfected by hand with individual siRNA duplexes and pools (both at 10 nM final concentration) in 384 well plates (Corning Costar #3712). 24 hours after transfection, cells were imaged to determine initial cell density. Following removal of the transfection mix, media containing either 150 nM of taxol (Paclitaxel) or 300 nM of nocadazole was added to the transfected cells. Cells were then incubated for an additional 48 hours and imaged for phenotypic changes at 24 hour intervals. The images generated were scored by visual inspection.

## Results and Discussion

### Concept of the screen

Our goal was to find proteins that, when their levels were reduced, made cells either more, or less, sensitive to small molecule inhibition of Kinesin-5. We hoped to find proteins that modified cell responses to the drug at the level of mitotic arrest, and also at the level of apoptosis following mitotic arrest, though in practice our screen was more effective for finding the former. The concept was to pre-treat cells with siRNAs targeting one protein, then to add drug at either a low concentration, scoring for enhancement of the drug effect, or at a high concentration, scoring for suppression. We elected to omit a no drug arm to save on reagents, and distinguished true enhancers from siRNAs that scored without drug in follow-up assays. The drug response plays out over several days in unsynchronized cells. At ∼24 hours after adding drug there is a peak in mitotic arrest, and at ∼48 hours a peak in apoptosis [8], [9]. We therefore sought an assay that allowed scoring the response at multiple time-points.

### Strategy for Assay Development

We chose high content cell imaging to read out our screening assay, an information rich method that facilitated monitoring of multiple phenotypes. We then considered whether to perform a fixed endpoint or time-lapse assay. Typically, an imaging screen for mitotic phenotypes would be performed as a fixed endpoint assay in microplates with immunofluorescence staining for DNA and indicators of mitotic arrest and perhaps also apoptosis [20]. The advantage of this method lies in ease of image analysis; with antibodies to different cell-cycle states, classification of cell phenotypes is straightforward. However, fixed endpoint imaging would limit our ability to quantify both the mitotic arrest and apoptosis responses, since they are separated by ∼24 hours. Other potential drawbacks of a fixed endpoint immunofluorescence assays include the cost of antibodies as well as potential loss of weakly adherent mitotic cells and distortion of cellular structures during fixation. Hence, a live cell time-lapse assay is, in principle, ideal for characterizing the effects of siRNAs that perturb cell division. However, time-lapse imaging across a whole genome screen is technically challenging. One approach was a miniaturized RNAi delivery system, in which cells were plated onto individually spotted siRNA transfection mixes, resulting in localized solid-phase transfection [14]. However, this approach required specialized instrumentation and time-consuming analysis techniques, and generated enormous amounts of data.

We developed a compromise strategy, intermittent live cell imaging. By imaging the same wells at several time points, we benefited from the ability to score the same wells immediately before drug, during peak mitotic arrest (∼24 hours in drug) and peak apoptosis (∼48 hours in drug), but can still use relatively standard image collection and analysis methods developed for fixed cells. This sparse time-lapse strategy should be useful for any assay where cell responses play out over a few days.

HeLa cells were chosen for the assay because they are easily transfected and a line expressing Histone H2B-GFP was available (Kanda et al., 1998, kind gift of Randy King, HMS). The cell phenotypes we needed to score have unique nuclear morphologies (interphase nuclei vs. mitotic chromosomes). Recent advances in computational methods have proven effective at using a single nuclear channel to quantify these phenotypes [21], [22]. One disadvantage of H2B-GFP is that the expression level is not constant from cell to cell, so intensity measures cannot be readily compared between cells, as they can for DNA staining with DAPI or Hoechst.

### Image analysis

Figure 3 shows examples of our image analysis methods. A cell in interphase (Fig. 3A) displays a relatively weak and even fluorescence signal resembling an ellipse with a smooth boundary. In bipolar mitotic spindles (Fig. 3B), condensed chromosomes line up in a metaphase plate, displayed as bright oblong objects under the microscope. Cells arrested in mitosis from treatment with K5I (Fig. 3C) or knockdown of certain siRNAs, have a characteristic monopolar spindle phenotype as previously reported [5]. The strong fluorescent signal arises from chromosomes condensed but scattered from the center of the cell, sometimes forming a ring.

The image analysis program CellProfiler Analyst [22] was used to perform machine learning based iterative training to determine the top 30 descriptors for each nuclear phenotype. For example, a monopolar mitotic cell displayed a characteristic spotted texture due to misaligned chromosomes. This property is quantified by top descriptors such as small form factor (defined as 4*pi*Area/Perimeter^∧^2), due to the rough edge resulting from chromatin radiating from the cell center, and large standard deviation of intensity, caused by chromosome condensation and overlap in the z-dimension.

Interphase nuclei display relatively uniform intensities, a property that allows straightforward detection by the descriptors with 95% accuracy. While larger intensity variations are observed between monopolar cells due to their slightly elevated focal plane, the accuracy of the descriptors is between 75–90% depending on image quality. Although bipolar mitotic spindles were another major phenotype observed in the screen with highly accurate descriptors, its classification was omitted due to uncertainty introduced by the relatively short duration of this phenotype.

Some cell density variation was observed after cells were plated with the Matrix WellMate. The distribution followed a Gaussian distribution slightly skewed to the right. The cell count cut-off for the step function in our formula to calculate Enhancer/Suppressor scores was set to 100, which excluded 251 (1.2%) and 362 (1.7%) low cell count wells from the enhancer and suppressor arms of the whole genome respectively.

To classify the phenotypes produced by each siRNA and to rank genes for further follow-up, data from CellProfiler were used to calculate Enhancer or Suppressor scores for each siRNA as described in detail in Materials and Methods. Based on these scores, the siRNAs were then prioritized for follow-up work. For secondary screening, we decided to follow up on genes in the top 2% of the Enhancer score list and bottom 2% of the Suppressor score list. Visual inspection was conducted on the images corresponding to these genes to eliminate false positives resulting from technical issues such as variation in focusing and/or exposure time.

### Enhancer Screen – Low Kinesin5 Inhibitor Concentration

Twenty-four hours after transfection, 60 nM of Kinesin-5 inhibitor was added to the cells to screen for siRNA enhancers of K5I activity. Based on pilot experiments, K5I administered at 60 nM induced no obvious increase in monopolar spindles. As shown in Figure 4, siRNAs that caused an increase in the number of monopolar spindles were easily identified. Cells treated with 60 nM K5I alone showed little difference to untreated cells (Figure 4A, B), while a hit phenotype demonstrated extensive monopolar metaphase arrest followed by apoptosis at subsequent time points (Figure 4E, F). To save time and money, we did not run a no-drug arm in the primary screen. Thus, many of our preliminary “enhancers” were expected to be siRNAs that cause mitotic arrest even in the absence of drug. These were distinguished from true enhancers in secondary screens, where we compared siRNA effects in low drug and zero drug.

**Figure 5:**
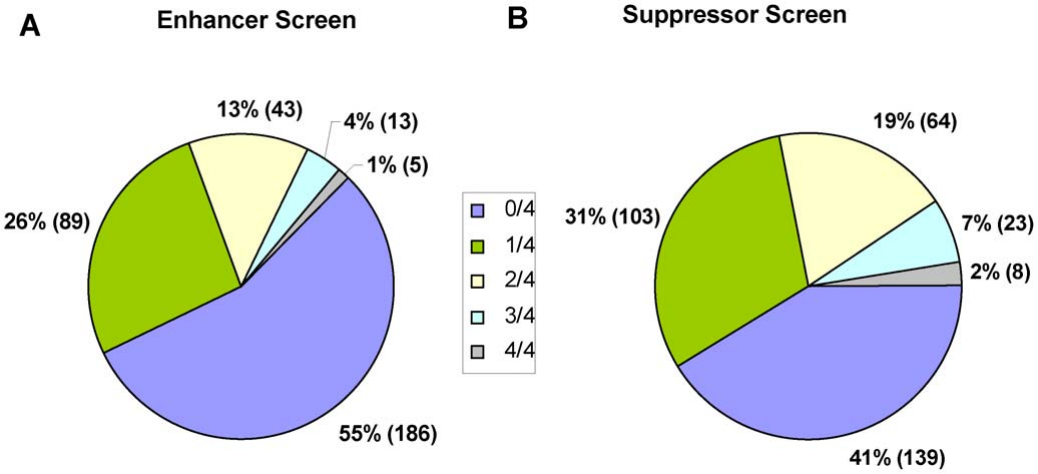
Breakdown of results for individual siRNA duplexes from preliminary hits for the K5I enhancer (A) and suppressor (B) screens. The slices of each pie chart indicate the percent of genes for each screen for which 4/4, 3/4, 2/4, 1/4, or 0/4 of the individual siRNA duplexes reproduced the enhancer or suppressor phenotype observed for the corresponding siRNA pool. For the enhancer screen, 4 individual siRNA duplexes for each of 336 siRNA pools that scored as hits in the primary screen were tested in the same assay as used for the primary screen. For the suppressor screen, the individual duplexes for each of 337 siRNA pools were tested.

A primary screen of 21,121 siRNA pools targeting most genes in the human genome identified 336 (1.6%) siRNAs that caused a significant increase in monopolar mitotic arrest in 60 nM K5I. Each pool targeted one gene and contained 4 individual siRNA duplexes. To confirm the K5I enhancer phenotypes, all four individual siRNAs from each preliminary screening positive pool were re-tested in the original assay, in duplicate. We found that at least one of four of the siRNA duplexes recapitulated the primary screen phenotype in 150 (45%) of the 336 pools followed-up in this way. Sixty-one (18%) of the positive pools confirmed with at least 2 out of 4 duplexes scoring per pool (Figure 5A, see Table S1 for the gene list).

To identify and eliminate siRNAs that arrest cells in mitosis independent of low-dose K5I treatment and are thus not true K5I enhancers, we monitored siRNA-induced mitotic arrest in the absence of drug for 90 of the 150 genes for which at least one out of four individual duplexes were shown to enhance K5I activity. We chose these 90 siRNAs based on their relatively strong enhancer phenotypes. siRNAs for 38 of the 90 genes were found to cause arrest of cells in mitosis in the absence of K5I treatment (see Table S1 for details). We also tested individual siRNA duplexes and pools for these 90 genes with a second, structurally diverse Kinesin-5 inhibitor from Merck Serono, used at sub-threshold concentrations, and found very similar results to our primary K5I results, confirming the specificity of the enhancer effect with respect to drug mechanism: for 87 out of 90 genes at least one siRNA duplex scored positive with both K5I inhibitors (30 out of 90 had one common positive siRNA duplex, 35 out of 90 had two common positive duplexes, 18 out of 90 had three common positive duplexes, and 4 out of 90 had four common positive duplexes.

Among the genes targeted by siRNAs that score as K5I enhancers, we found numerous genes involved in regulation of mitosis and cytokinesis. Some of these, including PLK1, STK6 (Aurora A Kinase, AURKA), SGOL1 (shugoshin), CDCA5 (sororin), CKAP5 (cytoskeleton associated protein, CH-TOG), and KIF11 (the target of our test drug), caused some increase in the fraction of cells in monopolar mitotic arrest with siRNA knockdown alone. These are all proteins known to play essential roles in mitosis, which, if completely knocked down, should on their own cause very strong mitotic arrest. Thus, while they score as K5I enhancers in the screen, they are better thought of as essential mitotic proteins. We surmise that the siRNA knockdown is incomplete in most cases, and that low drug concentrations have an effect on mitotic phenotype that is additive with partial loss of protein. This consideration complicated analysis of the enhancer siRNAs, and we therefore prioritized K5I suppressor siRNAs for further analysis. A complete list of the K5I enhancer siRNAs identified in our screen is provided in Table S1.

### Suppressor Screen – High Kinesin5 Inhibitor Concentration

**Figure 6:**
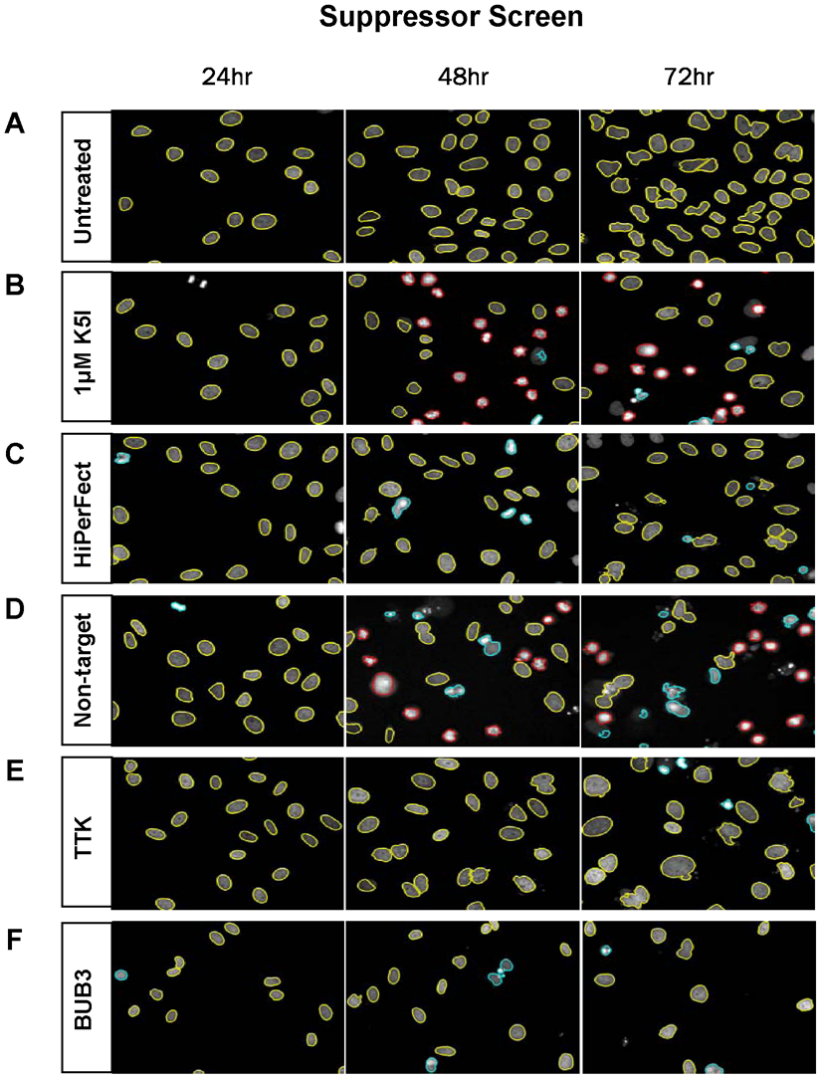
Sample strong hit phenotypes from the K5I Suppressor screen. Nuclei from each time point are color-coded to illustrate their CellProfiler classifications: yellow = interphase, red = monopolar, blue = unclassified objects. As illustrated in Fig. 2, transfection occurred at T = 0. (A) untreated HeLa H2B-GFP cells, (B) untransfected cells treated with 1 uM K5I, and (C) mock transfected cells treated with HiPerFect transfection reagent and no K5I. In panels D–F, 1 uM K5I was added to cells 24 hours after transfection with (D) non-targeting siRNA, or siRNAs directed against (E) TTK or (F) Bub3.

For the suppressor screen, transfected cultures were treated with 1 uM K5I, which causes 80–90% of cells to accumulate in monopolar mitosis after 24 hours of drug treatment (Figure 6B). After 48 hours in high concentrations of K5I (72 hours after transfection), cell density begins to drop due to subsequent apoptosis [8]. In our screen, a suppressor hit phenotype was identified by decreased monopolar mitotic arrest after 24 hours in K5I (48 hours after transfection), or an increased cell survival ratio after 48 hours in K5I (72 hours after transfection). Both phenotypes could correspond to decreased mitotic arrest, and/or decreased or earlier apoptosis (Figure 6E, F).

Primary screening revealed 337 (1.6%) potential suppressor siRNA pools. In secondary screening of individual siRNA duplexes (4 duplexes/siRNA pool), 198 (59%) of the pools that scored as positive in the primary screen retested in duplicate with at least 1 duplex scoring for each pool, and 95 (28%) of the pools retested with at least 2 out of 4 duplexes scoring per pool (Figure 5B, see Table S1 for the gene list).

False positives due to “off-target” effects of RNAi are a notorious problem in siRNA screens in human cells with current technology, and no completely satisfactory method is yet available to eliminate them. We chose the simple expedient strategy of prioritizing the 31 genes for which 4/4 or 3/4 individual siRNA duplexes demonstrated the K5I suppressor phenotype in the secondary screen. This should enrich for true positives, though it will no doubt eliminate many. We added one other gene, KATNB1 (a subunit of the microtubule severing enzyme Katanin) that scored with 2/4 duplexes, because of its interesting function.

**Figure 7:**
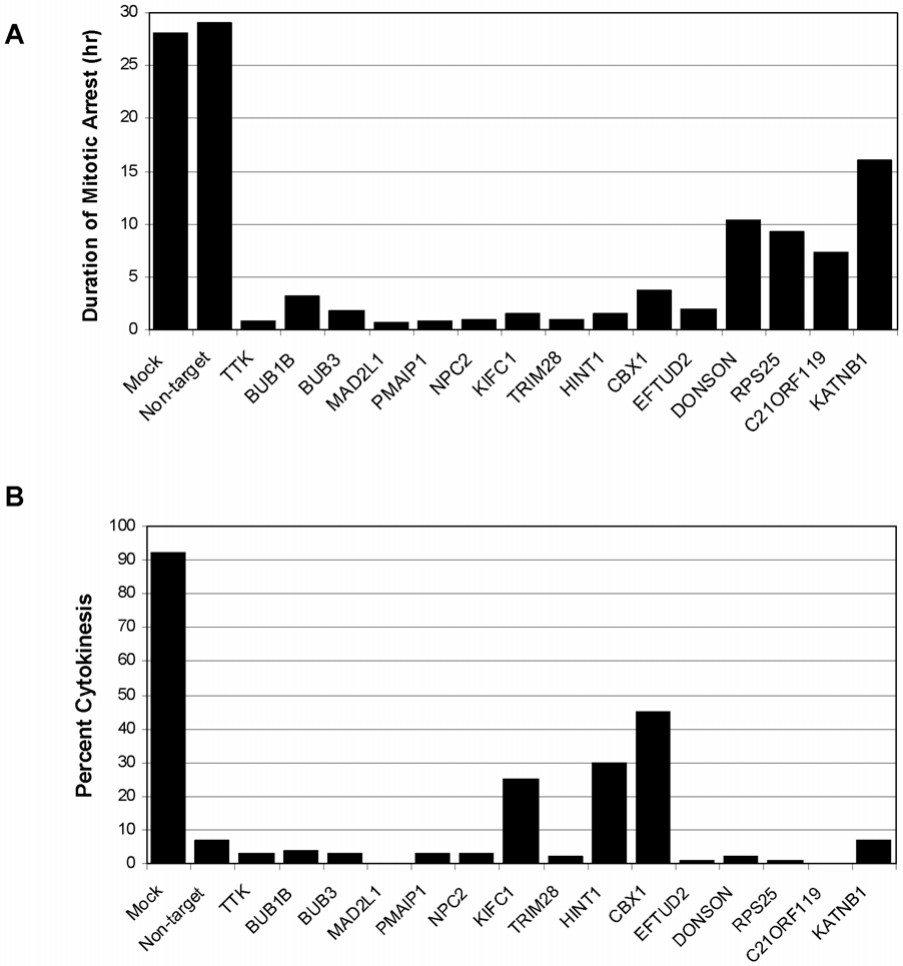
Results of time-lapse experiments to examine duration of mitotic arrest and percent of cells entering cytokinesis after treatment with K5I suppressor siRNAs. HeLa H2B-GFP cells were transfected with siRNA pools and individual siRNA duplexes directed against the indicated genes or with a non-targeting control siRNA. “Mock” cells were treated with HiPerFect transfection agent in the absence of siRNA. 24 hours after transfection, K5I was added to all of the samples, so that the final inhibitor concentration was 1 uM. Cells were imaged at 10 minute intervals for 48 hours on an automated inverted fluorescence microscope and automated focus. 50–100 cells from each condition were scored by visual inspection for (A) the average duration of mitosis in the population, and (B) the percent of cells going through cytokinesis upon exit from mitosis. For each of the genes shown, both the siRNA pool and the individual siRNA duplexes were observed to cause the same phenotypes, but only data for the siRNA pools is graphed here.

**Figure 8:**
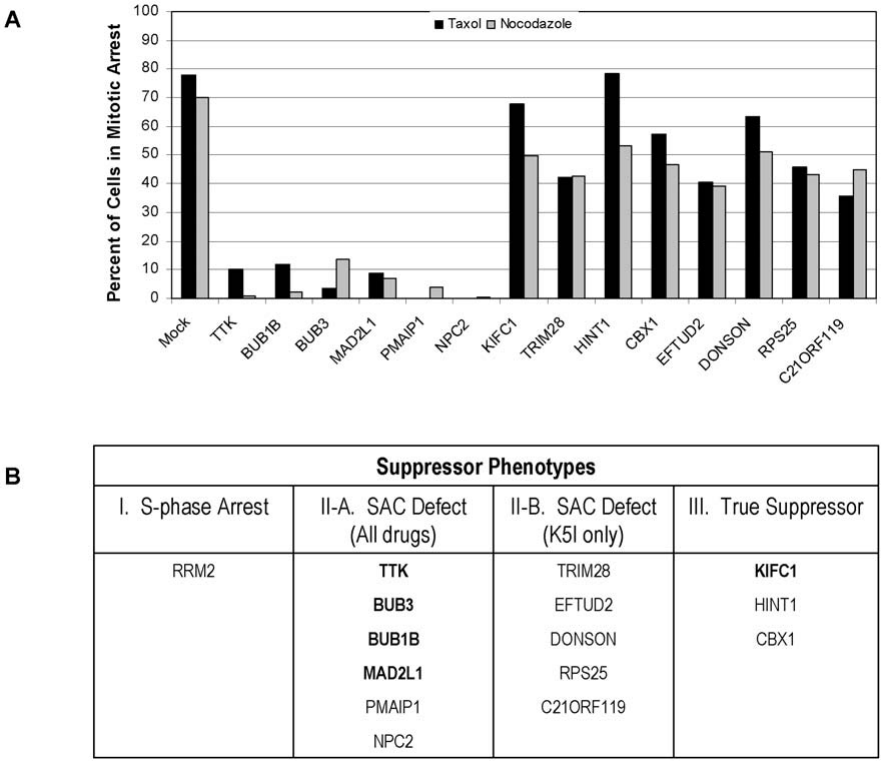
Drug specificity and final phenotypes. A: Drug specificity tests. HeLa H2B-GFP cells were transfected with siRNA pools as indicated, or “Mock” transfected in the absence of siRNA. 24 hours after transfection, medium containing either 150 nM of taxol or 300 nM of nocadazole was added to all of the samples. Cells were then incubated for an additional 24 hours and imaged for phenotypic changes. The pools were scored visually for cells in mitotic arrest, and this value was quantified for the pool by counting >100 cells. (B) Phenotypic categorization of hits on the basis of data in Figure 7 and 8A. Category I) failure to enter mitosis, II) brief mitotic arrest without cytokinesis, and III) brief mitotic arrest with cytokinesis (true suppressors). Shown in bold are 5 expected genes, 4 known SAC genes and KifC1, which is a known true suppressor of loss of Kinesin-5 function [28].

The mitotic arrest and apoptosis phenotypes of high K5I could potentially be suppressed by multiple mechanisms, and we used time-lapse imaging to classify the 32 prioritized primary hits by phenotype. HeLa H2B GFP cells were transfected with the four individual duplexes, and also the pool of 4, at 15 nM total siRNA, a lower concentration than the primary screen to further select for on-target effects. 24 hours later 1 µM K5I was added, and images were collected every 10 minutes for 48 hours using an automated inverted microscope fitted with an incubation chamber. Movies were scored by visual inspection for three aspects of phenotype: entry into mitosis, time in mitosis, and whether cells divided after exit from mitosis, or simply flattened out due to failed cytokinesis. Visual inspection was used to identify genes for which at least 2 siRNAs had a strong phenotype (15 genes), and for preliminary classification. The data in Figures 7 and 8 corresponds to these strong phenotype genes.

By visual inspection, we were able to divide our hits into three phenotypic classes: I) failure to enter mitosis, II) brief mitotic arrest without cytokinesis, and III) brief mitotic arrest with cytokinesis. We will refer to category III siRNAs as “true suppressors.” The only strong hit in category I was RRM2, a subunit of ribonucleotide reductase that is essential for progression through S-phase. Blocking cells in S-phase was expected as a mechanism for lowering the mitotic index in K5I. We expected many such hits, but only RRM2 passed all our prioritization criteria, probably because cells would have had to arrest within ∼12 hours of addition of siRNA treatment, which would select for very unstable and/or dose-sensitive proteins. To measure the penetrance of the siRNA phenotypes for categories II and III, we scored the average duration of mitosis arrest compared to control siRNA (Figure 7A), and the fraction of cells leaving mitosis that underwent cytokinesis (Figure 7B). These data were collected from the movie that showed the strongest phenotype for the 4 single siRNAs or the pool; in many cases the pool was the strongest, and in all cases more than one movie showed the same phenotype. All 15 genes exhibited mitotic arrest durations of less <16 hours for either the pool or the single strongest oligo, and for most of them it was <4 hours, as compared to >20 hours in control siRNA. Scoring for the fraction of cells that underwent cytokinesis approximately divided the genes into two groups. Using 10% cytokinesis as an arbitrary dividing line, siRNAs for 3 genes rescued cytokinesis (true suppressor phenotype, category III), while 12 suppressed mitotic arrest, but did not rescue cytokinesis (category II).

### Drug specificity

To further categorize the genes that shortened mitotic arrest, we tested their ability to suppress mitotic arrest caused by Paclitaxel (which blocks microtubule dynamics) and nocodazole (which depolymerizes microtubules). Both drugs arrest cells in mitosis by activating the spindle assembly checkpoint (SAC), like K5I. We tested all the genes in Figure 7, except KATNB1. HeLa H2B GFP cells were transfected with individual siRNAs and the pool of 4, and 24 hours later we added 150 nM taxol or 300 nM nocodazole. Twenty-four hours after that, cells were fixed, and the fraction arrested in mitosis under these conditions was scored by visual inspection (Figure 8A). We quantified only the pool, but visually confirmed that at least 2 of the individual oligos scored with the same phenotype in all cases. In Paclitaxel-treated wells where the mitotic arrest was suppressed, we observed a large percentage of cells in interphase with multiple micronuclei. This phenotype is characteristic of slippage out of taxol-induced arrest [23]. This experiment appeared to cleanly divide the genes into two classes, those that strongly suppressed both taxol and nocodazole in addition to K5I, and those that only suppressed K5I. All the annotated SAC genes suppressed all the drugs, as expected. Two other genes scored with this phenotype, PMAIP1 and NPC2, and these were also notable for very short mitotic arrest durations (Figure 7A). The 3 true suppressors (category III) failed to suppress taxol and nocodazole. A number of category II genes failed to suppress taxol and nocodazole, including all those that less effectively suppressed K5I mitotic arrest as scored by duration (Figure 7A), but also two that strongly suppressed K5I, TRIM28 and EFTUD2. On the basis of this extra data, we divided category II into two, SAC defect (all drugs) and SAC defect (K5I) (Figure 8B).

### Mechanistic implications

An important caveat in discussing any of the new genes in Figure 8 is the question of specificity. Although we tried to select for on-target effects by requiring at least 3 out of 4 oligos to score, we suspect some of our hits may still be false positives. Further work, including monitoring protein levels after knockdown and rescue with RNAi-resistant transgenes, is required to more rigorously test their specificity.

In the SAC defect (all drugs) category, we recovered 4 known SAC genes: TTK (Mps1 kinase), BUB3, BUB1B, and MAD2L1. We missed a few other well-annotated SAC genes, including MAD1, presumably because the knockdown was insufficient in our time window. Two other genes scored with the same phenotype, NPC2 and PMAIP1. NPC2 is an endosome protein implicated in cholesterol transport [24], and PMAIP1 (also known as NOXA) is a BH3-only pro-apoptotic protein that stimulates apoptosis by interacting with Bcl2 related proteins [25]. Because of its possible role in linking mitotic arrest to apoptosis, we performed limited follow-up work on PMAIP1/NOXA. Different siRNA oligos for this gene exhibited different strengths of phenotypic effect as suppressors of mitotic arrest. In addition to knocking down PMAIP1 protein levels, we noted that the two siRNAs (#2 and #3) that inhibited mitotic arrest most effectively also caused partial knockdown of Mad2 protein levels (Figure S1), though they did not affect expression of another checkpoint protein BubR1 (data not shown). Thus it is possible that the K5I suppressor effect of the PMAIP1 siRNA reagents are due to an off-target knockdown of Mad2 by the PMAIP1 siRNAs. Consistent with this, the seed regions of the PMAIP1 siRNA#2 and #3 each share sequence identity with different 7 nucleotide stretches of the MAD2L1 3′ UTR mRNA sequence (RefSeq NM_002358.3). siRNA seed region matches to 3′ UTR regions are associated with RNAi off-targets [26], [27].

The SAC defect (K5I only) category was unexpected, and the gene annotations provide little in the way of mechanistic clues. DONSON is, by sequence, the probable ortholog of the Drosophila gene humpty dumpty, which has been implicated in control of DNA replication [26]. RPS25 is a ribosome subunit, and thus might be a false positive. EFTUD2 is annotated as a spliceosome component. Knocking down these proteins appears to suppress SAC activation by K5I, but not by taxol or nocodazole, yet they do not rescue spindle bipolarity, at least by the criterion of promoting cytokinesis. This exclusivity for K5Is could be the result of a more weakly activated SAC by K5I, or that the SAC is activated in a different way, that requires extra proteins to block the cell cycle relative to taxol and nocodazole activation of the SAC. It is also interesting that K5Is do not target the microtubules themselves, perhaps resulting in SAC-dependent arrest that is unique at the molecular level compared to microtubule targeting drugs.

Category III, the true suppressors, were particularly interesting from a spindle assembly perspective. This phenotype requires that the cell assemble a normal bipolar spindle even though Kinesin-5 is inhibited, or alternatively, this class might by due to increased drug efflux or metabolism. KIFC1 (also known as HSET), a mitotic kinesin that works opposite to Kinesin-5, was expected with this phenotype, since previous work has demonstrated that loss of KIFC1 function can rescue spindle bipolarity when Kinesin-5 function is compromised [28]. The strongest suppressor was CBX1, a heterochromatin protein. 45% of cells that entered mitosis underwent cytokinesis in cells treated with this siRNA pool in the presence of 1 µM K5I (Fig. 7B). This value can be compared to <1% cytokinesis in K5I alone, and 3% for TTK siRNA (a strong Category II hit) in K5I. We tentatively speculate that CBX1 is required for some aspect of kinetochore or chromokinesin function that normally helps pull the two half-spindles together. When this function is absent, the forces that collapse the spindle in K5I are reduced. Another strong suppressor, HINT1, is an enzyme with phosphoramidase activity that is a tumor suppressor, and has been implicated in DNA damage repair [28]. Perhaps this protein also influences the spindle via effects on chromatin structure.

### Summary

Our intermittent imaging approach was effective for identifying siRNA enhancers and suppressors of K5Is that act at the level of spindle assembly and mitotic arrest, and should be useful for other cell cycle related screens. It was less effective at identifying genes that influence apoptotic responses, perhaps because we were not able to develop a specific score for apoptosis using H2B-GFP imaging. Characterization of siRNAs that score as K5I enhancers was complicated by the fact that many of those siRNAs had some mitotic phenotype on their own, in addition to acting as enhancers of drug action. However, the enhancer siRNA hit list is strongly enriched in genes with mitotic functions, and may reveal other genes not previously implicated in spindle bipolarization. Our K5I suppressor siRNAs broke fairly cleanly into 4 phenotypes (Figure 8B). Current RNAi technology is sufficiently prone to false positives that we make no claims as to specificity of our hits. Even genes for which 4/4 individual siRNA duplexes scored as hits must be viewed with suspicion until confirmed by other methods. For example, the possibility that PMAIP1 scored as a hit due to off-target knockdown of Mad2 levels (Figure S1) is a case in point. We also acknowledge that we surely missed many genes as false negatives Presumably, our screen selected for proteins that turn over rapidly and/or whose effects on their pathway are highly dose-sensitive. Still, we recovered about half of the well-documented SAC genes, and the only known true K5I suppressor gene (KIFC1), in the expected phenotypic categories. Thus, we are confident that at least some of our others hits are worthy of further exploration, and will open up new avenues of research into spindle organization and the SAC.

## Supporting Information

Figure S1 (0.15 MB TIF)

Table S1 List of genes with at least one out of four oligos confirmed in the same assay as used for the primary screen.
(0.06 MB XLS)
