# Supplementary figures and images for: An Intermittent Live Cell Imaging Screen for siRNA Enhancers and Suppressors of a Kinesin-5 Inhibitor
Source: PLoS One. 2009 Oct 5;4(10):e7339. doi: 10.1371/journal.pone.0007339 (PMC2752188; doi:10.1371/journal.pone.0007339)

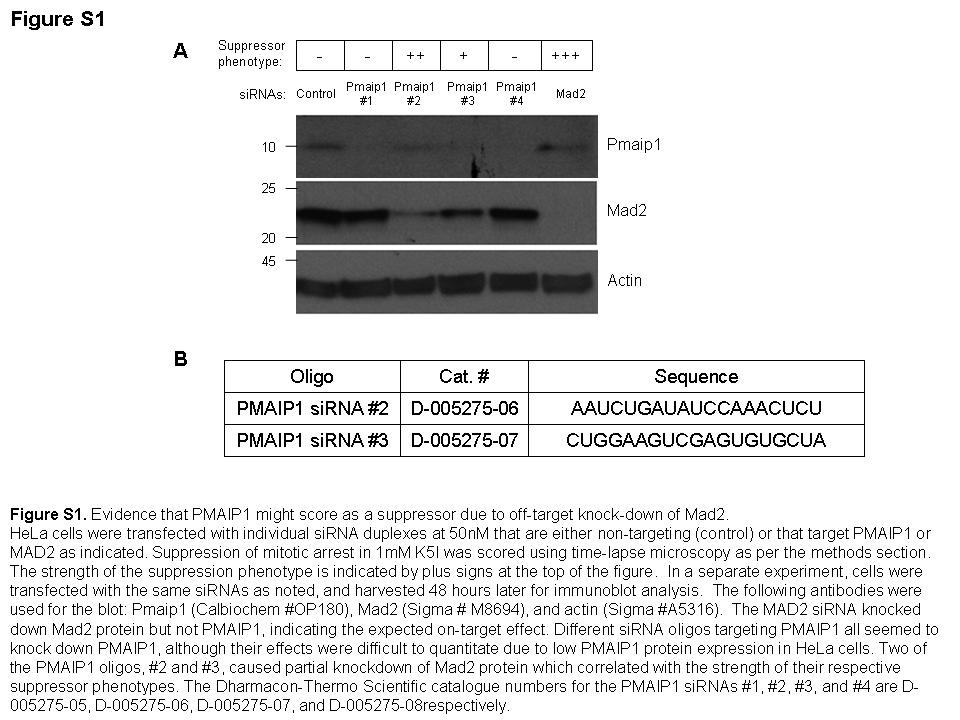

Supplement: Figure S1 — (0.15 MB TIF) [file pone.0007339.s001.tif]
